# Solid pseudopapillary tumor of the pancreas (Frantz’s tumor): two case reports and a review of the literature

**DOI:** 10.1186/s13256-015-0752-z

**Published:** 2015-11-20

**Authors:** Żaneta Słowik-Moczydłowska, Michał Gogolewski, Sadeq Yaqoub, Anna Piotrowska, Andrzej Kamiński

**Affiliations:** Department of Pediatric Surgery, Medical University of Warsaw, Marszałkowska 24, Warsaw, Poland

**Keywords:** Frantz’s tumor, Pancreatic cancer, Pancreatic tumor, Pancreatoduodenectomy, Solid pseudopapillary tumor

## Abstract

**Introduction:**

Solid pseudopapillary tumor of the pancreas is extremely rare in children; it usually occurs in young women between 18 and 35 years of age. It comprises less than 3 % of pancreatic tumors. It is of low malignancy; however, it may be locally aggressive. Surgical resection is the treatment of choice and its prognosis is excellent.

**Case presentation:**

Two Caucasian girls, 15 and 12 years of age were diagnosed with tumor of the pancreas. The first patient had severe abdominal pain. In the second case the tumor was asymptomatic, detected incidentally during ultrasound. Computed tomography confirmed pancreatic mass. In the first case, apart from the tumor located in the head and the body of her pancreas, focal change in her right kidney was found, which was an indication to biopsy that confirmed solid pseudopapillary tumor. In the second patient the tumor was located in the body of her pancreas, with portal vein occlusion and well-developed collateral circulation. In the first patient a pancreatoduodenectomy (Traverso-Longmire) was performed; there was no mass in her right kidney. In the second case, distal pancreatectomy and splenectomy were performed. In both cases histopathology revealed solid pseudopapillary tumor resected radically. Our first patient’s postoperative course was uneventful. In the second case, her postoperative course was complicated by necrosis of the remaining pancreatic head that needed pancreatoduodenectomy. Follow-up at 28 and 26 months revealed no evidence of tumor recurrence or metastases on magnetic resonance imaging.

**Conclusions:**

Typical radiological appearance of solid pseudopapillary tumor is an indication for surgery. The treatment of choice is tumor resection with sparing of pancreatic tissue. In one of our two cases we performed a preoperative biopsy because of an uncharacteristic mass in her right kidney. In our second patient, necrosis of her spared pancreatic head meant that we could not preserve pancreatic tissue. Our whole diagnostic process, treatment and possible complications analysis should be of interest and noteworthy not only to surgeons as the treatment of choice is radical resection, but also to pediatric oncologists because of differentiation from other pancreatic tumors in children.

## Introduction

Solid pseudopapillary tumor of the pancreas (SPT, Frantz’s tumor) is very rarely diagnosed in children. The entity was first described in 1959 by pathologist Virginia Kneeland Frantz and in 1996 reclassified by the World Health Organization (WHO) [1–3]. It comprises only 0.2 to 2.7 % of all tumors of the pancreas. Up to 2014 there were approximately 900 well-documented cases of pancreatic SPTs with only a small minority of them concerning children [4–7]. It occurs predominantly in young women between 18 and 35 years of age. The clinical manifestation of this disease is usually a slowly growing abdominal mass with or without abdominal pain. It is of low malignant potential; however, some cases may be locally aggressive and infiltrative, with metastases to the liver, lung and skin [4]. The pathogenesis is still unknown [8]. On histological examination the tumor is a solid mass with pseudopapillary and pseudocystic structures with rich microvasculature in various proportions. Surgical resection is the treatment of choice, and its prognosis is excellent, with 10-year survival approaching 100 %.

The aim of this study is to analyze symptoms, diagnosis, treatment and long-term outcomes of two adolescent girls treated for SPT of the pancreas.

## Case presentation

### Case 1

**Fig. 1:**
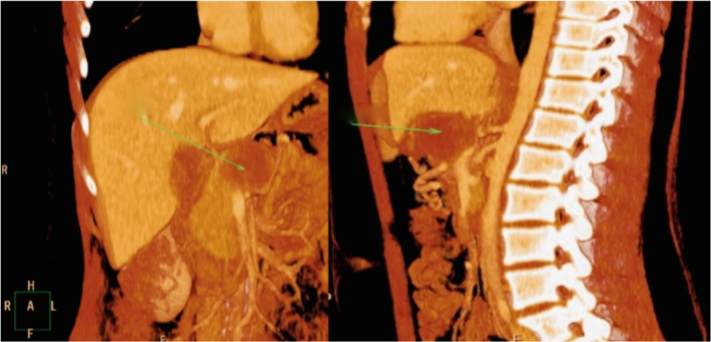
Computed tomography scans showing the presence of the tumor located in the head and the trunk of the pancreas. The tumor measured 4.8 × 4.2 × 5 cm^3^ (*arrows*)

**Fig. 2:**
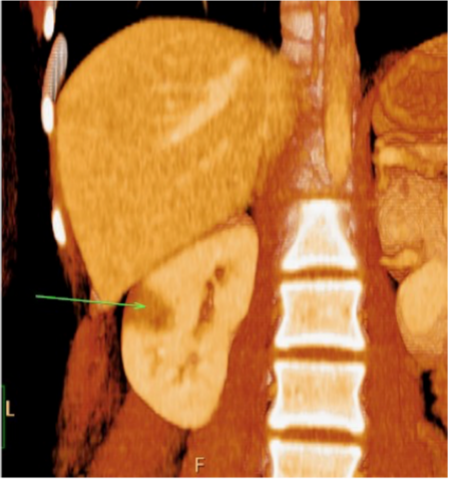
Computed tomography scan showing additional mass. The mass (approximately 2 cm) was detected in the upper pole of the right kidney (*arrow*)

**Fig. 3:**
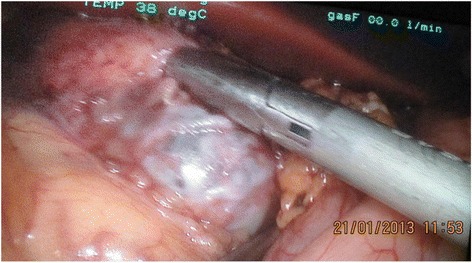
Laparoscopic biopsy of pancreatic mass

**Fig. 4:**
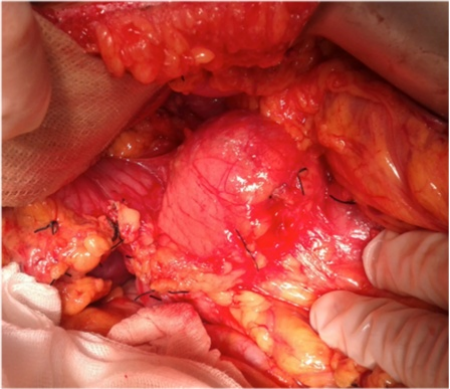
Encapsulated tumor approximately 6 cm in diameter located in the head and the trunk of the pancreas

**Fig. 5:**
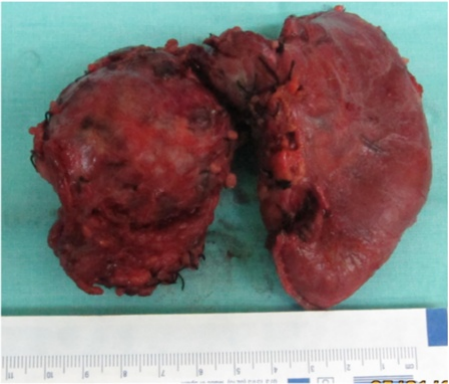
Specimen of resected pancreatic head with the tumor and the duodenum

**Fig. 6:**
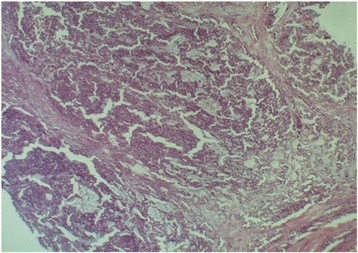
Histopathology confirming solid pseudopapillary tumor (HE×100)

A 15-year-old Caucasian girl was admitted to our department with severe epigastric pain over 2 days with a temperature of 38 °C. In laboratory tests slightly elevated white blood cells (WBC) level (12 × 10^3^) and C-reactive protein (5.3 mg/dl) were found. An ultrasound (USG) examination and computed tomography (CT) scans showed the presence of a tumor located in the head and the trunk of her pancreas (4.8 × 4.2 × 5 cm^3^; Fig. 1). Another mass (approximately 2 cm in diameter) was detected in the upper pole of her right kidney (Fig. 2). The tumor markers carcinoembryonic antigen (CEA), carbohydrate antigen (CA19-9), and alpha-fetoprotein (AFP) were all within the normal range. A bone marrow biopsy revealed no abnormalities. A laparoscopic biopsy of the pancreatic mass (Fig. 3) showed solid pseudopapillary tumor of the pancreas. Laparoscopy was converted to laparotomy that showed an encapsulated tumor of approximately 6 cm in diameter located in the head and the trunk of her pancreas (Fig. 4). An inspection of her right kidney showed no tumor. Pancreatoduodenectomy (Traverso-Longmire) was performed (Fig. 5) with spleen preservation. Histopathology confirmed completely resected SPT (Fig. 6). Her postoperative course was uneventful. There was no adjuvant therapy. Renal scintigraphy and magnetic resonance imaging (MRI) after 4 months revealed no metastases. A follow-up 28 months later showed neither signs of tumor recurrence nor endocrine and exocrine insufficiency of the pancreas.

### Case 2

**Fig. 7:**
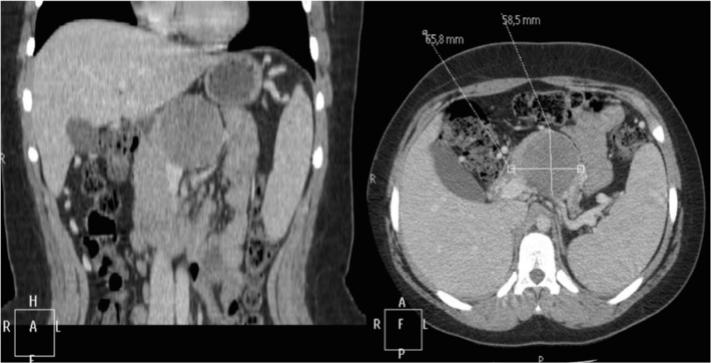
Computed tomography scans showing a cystic and solid mass 5.2 × 5.5 × 5.2 cm^3^ arising from the trunk of the pancreas

**Fig. 8:**
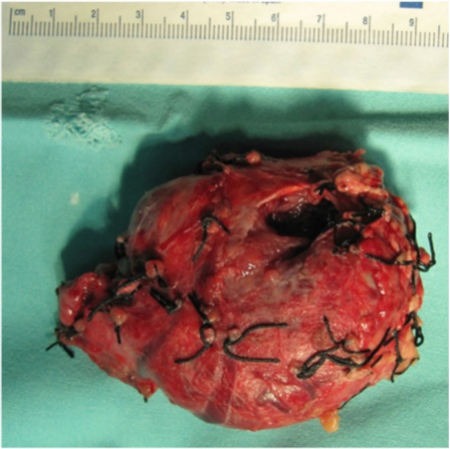
Solid 6 × 6 cm in diameter tumor of the trunk and the tail of the pancreas

**Fig. 9:**
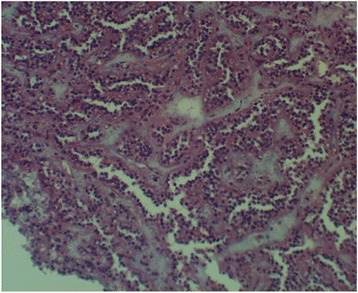
Histopathology confirming completely resected Frantz’s tumor

A 12-year-old Caucasian girl was admitted to our department after a mass in her pancreas was incidentally revealed during USG. CT scans showed a cystic and solid mass 5.2 × 5.5 × 5.2 cm^3^ arising from the trunk of her pancreas (Fig. 7). The tumor was compressing her superior mesenteric vein and infiltrating her splenic vein suggesting local invasion. There was no evidence of distant metastases or abnormalities in laboratory tests. Laparotomy showed a solid tumor 6 × 6 cm in diameter of the trunk and the tail of her pancreas (Fig. 8), as well as portal vein thrombosis with collateral circulation. A complete tumor resection including left pancreatectomy and splenectomy was performed. Histopathology revealed completely resected Frantz’s tumor (Fig. 9). Her postoperative course was complicated by necrosis of the pancreatic head thus Whipple procedure was performed. Histopathological examination of the pancreatic head specimen showed necrotic tissue without tumor cells. Her postoperative course was uneventful. No additional therapy was administered. MRI of her abdomen 4 months later revealed no metastases. A follow-up 26 months later showed no signs of tumor recurrence and no diabetes. Gastroscopy revealed I^0^ esophageal varices treated endoscopically.

## Discussion

SPT of the pancreas is extremely rare in children. It is usually found incidentally on radiologic examination for other reasons and there are no typical clinical signs and symptoms. In some patients the tumor is noticed because of chronic or acute abdominal pain. SPT is usually localized in the pancreatic head. Exocrine or endocrine insufficiencies have not yet been described. No specific tumor markers are known [4–9]. In one of our cases SPT was discovered incidentally and in the other because of acute abdominal pain. The localization of the tumors was head or trunk of the pancreas. In both cases there were no characteristic biochemical abnormalities, which is in accordance with reports from other authors. Awareness of the radiologic features of Frantz’s tumor is very important to establish an accurate diagnosis before operation and planned further surgery. The typical tools used to diagnose are abdominal USG and CT scan. SPT appears as a solid well-demarcated mass, usually heterogenous in echo texture, sometimes containing hypoechoic fluid-filled cystic areas in USG. CT scans show a heterogenous mass, often with peripheral contrast enhancement corresponding to the fibrous pseudocapsule [4–10]. In one of our cases the tumor was solid and in the other it was cystic and solid. Histological evaluation of SPT shows typically solid and pseudopapillary structures, intensive vascularization, or hemorrhagic pseudocystic structures in various proportions [11]. This picture was also seen in the pathohistological examination in our patients. Some authors advocate preoperative fine-needle biopsy (FNB) for distinguishing between benign and malignant lesions. It can guide surgical management, because a potentially benign tumor (for example, neuroendocrine, SPT) may be treated by local excision, in contrast to an adenocarcinoma, which would require a more extensive resection [12]. Other authors may not accept this because of the uncertainty in diagnosis and possible tumor cell spread. FNB, for instance, may not differentiate between pancreatoblastoma, the typical pancreatic tumor of young age, and Frantz’s tumor [9]. We performed a preoperative biopsy in Case 1 because of a suspected additional mass in her right kidney, which was not typical for SPT and could indicate non-Hodgkin lymphoma (NHL). In Case 2 the characteristic radiological appearance of Frantz’s tumor was sufficient for qualification for laparotomy and a preceding biopsy was not necessary. The low grade of malignancy of this tumor, and because the mass is usually surrounded by a dense fibrous capsule, led to complete resection with preservation of as much pancreatic tissue as possible. Local resection is therefore the therapy of choice. Distal pancreatectomy with or without splenic preservation can be performed for tumors in the trunk or tail of the pancreas, and pancreatoduodenectomy for tumors of the pancreatic head. Our first patient underwent a pancreatoduodenectomy (Traverso-Longmire) because of the tumor localization in the head and the trunk of her pancreas. In our second case, we could perform a left pancreatectomy because the tumor arose from the trunk and the tail of her pancreas. Control MRI showed tissue similar to remnants of the pancreatic tail which might explain normal levels of glucose. In this patient postoperative complication made the preservation of the pancreatic head impossible. Descriptions from case reports concerning incomplete resection of Frantz’s tumor between 1985 and 2008 show that despite the low malignant potential of SPT, median survival in patients who underwent incomplete resection was only 5.7 years. That is why complete resection of Frantz’s tumor is always justified, even at the price of difficult mutilating surgery [13]. This is the reason we should be searching for less radical options with complete resection of the tumor as described in literature central pancreatectomy of a 5 cm tumor located in the body of pancreas [14]. Up to now there has been no clear role for chemotherapy or radiotherapy in cases of malignancy, inoperable tumors or relapse. Reported cases and reviews from the literature where unresectable SPT was treated with chemotherapy and radiotherapy show benefits in a limited number of patients, such as the report of a case of a 14-year-old girl with unresectable Frantz’s tumor with a 7 × 7 cm diameter mass compressing her superior mesenteric vein with acute pancreatitis. After two cycles of gemcitabine a repeated CT scan showed significant interval decrease in the size of the pancreatic mass 1.5 × 1.0 cm without compression on her superior mesenteric vein and no evidence of pancreatitis. She was able to successfully undergo a pyloric-sparing Whipple procedure [15, 16]. Our two patients were not considered for preoperative chemotherapy or radiotherapy. In both cases the tumor was qualified as radically resectable. At present both patients remain without tumor recurrence.

## Conclusions

Typical radiological appearance of SPT is an indication for surgery. The treatment of choice in SPT is tumor resection with sparing of pancreatic tissue. We performed a preoperative biopsy in one of our two cases because of suspected additional mass in the right kidney. In the other case, SPT of the trunk and tail of the pancreas associated with portal vein thrombosis and collateral circulation meant that we could not preserve the pancreatic head. Control MRI revealed a small piece of tissue characteristic of the pancreatic tail above the upper pole of the left kidney, which could explain why an insulin supply was not needed during the postoperative course.

## Consent

Written informed consent was obtained from the patients’ legal guardians for publication of this case report and any accompanying images. A copy of the written consent is available for review by the Editor-in-Chief of this journal.

## Abbreviations

CT: computed tomography
FNB: fine-needle biopsy
MRI: magnetic resonance imaging
SPT: solid pseudopapillary tumor
USG: ultrasound
